# A multimodal travel route recommendation system leveraging visual Transformers and self-attention mechanisms

**DOI:** 10.3389/fnbot.2024.1439195

**Published:** 2024-11-26

**Authors:** Zhang Juan, Jing Zhang, Ming Gao

**Affiliations:** ^1^Fanli Business School, Nanyang Institute of Technology, Nanyang, Henan, China; ^2^Hospitality Management Department, Tourism College of Zhejiang, Hangzhou, Zhejiang, China

**Keywords:** multimodal travel recommendation, visual Transformer, self-attention mechanism, image and sequence fusion, deep learning

## Abstract

**Introduction:**

With the rapid development of the tourism industry, the demand for accurate and personalized travel route recommendations has significantly increased. However, traditional methods often fail to effectively integrate visual and sequential information, leading to recommendations that are both less accurate and less personalized.

**Methods:**

This paper introduces SelfAM-Vtrans, a novel algorithm that leverages multimodal data—combining visual Transformers, LSTMs, and self-attention mechanisms—to enhance the accuracy and personalization of travel route recommendations. SelfAM-Vtrans integrates visual and sequential information by employing a visual Transformer to extract features from travel images, thereby capturing spatial relationships within them. Concurrently, a Long Short-Term Memory (LSTM) network encodes sequential data to capture the temporal dependencies within travel sequences. To effectively merge these two modalities, a self-attention mechanism fuses the visual features and sequential encodings, thoroughly accounting for their interdependencies. Based on this fused representation, a classification or regression model is trained using real travel datasets to recommend optimal travel routes.

**Results and discussion:**

The algorithm was rigorously evaluated through experiments conducted on real-world travel datasets, and its performance was benchmarked against other route recommendation methods. The results demonstrate that SelfAM-Vtrans significantly outperforms traditional approaches in terms of both recommendation accuracy and personalization. By comprehensively incorporating both visual and sequential data, this method offers travelers more tailored and precise route suggestions, thereby enriching the overall travel experience.

## 1 Introduction

In recent years, with the improvement of living standards and the increasing demand for travel, efficiently recommending personalized travel itineraries has become an urgent problem to be addressed. Traditional travel route recommendation methods often rely on expert experience, which struggles to meet personalized needs and fails to handle large-scale and complex data effectively (Renjith et al., 2020). With the introduction of machine learning techniques, it has become possible to process big data more efficiently and provide accurate personalized recommendations based on users' historical behavior and preferences (Ji et al., 2020). Through the iterative optimization process of machine learning algorithms, the accuracy and efficiency of recommendation systems have been significantly improved, leading to a more satisfying user experience (Egli et al., 2020). Therefore, research on travel itinerary recommendations not only holds theoretical significance but also promotes its practical application.

Traditional travel recommendation methods primarily rely on symbolic AI and knowledge representation, usually implemented through expert systems that simulate human experts' decision-making processes. These systems can encode expert knowledge and provide clear explanations for each recommendation, such as the multi-agent knowledge system proposed by Lorenzi (2007). Another category of methods relies on predefined rules, exhibiting high determinism and reliability, which perform well in complex or dynamic travel scenarios. Gandhi et al. (2014) introduced a rule-based system for automated travel analysis, while Jiang and Dai (2024) presented a rule-based system framework for analyzing travel performance. Although these methods offer strong interpretability and transparency, they fall short in handling large-scale data and complex travel demands. Simulation computing techniques can predict and analyze travel behavior by constructing and running simulation models, but they are still insufficient for addressing complex, dynamic needs, and processing large-scale data (Gong et al., 2023; Khan et al., 2023).

To overcome the limitations of traditional algorithms in terms of adaptability and handling complex requirements, data-driven and machine learning-based algorithms have optimized recommendations by analyzing large volumes of user data and historical behavior, offering high accuracy and personalized recommendations. Decision tree-based methods have been widely applied for user classification and tourism recommendations. Kesorn et al. (2017) used the C4.5 decision tree algorithm to recommend travel regions for tourists, while Kbaier et al. (2017) proposed a personalized hybrid travel recommendation system that uses decision tree algorithms to recommend attractions based on user preferences. Random forest algorithms improve the stability and accuracy of recommendations by combining predictions from multiple decision trees (Li, 2024), and Support Vector Machines (SVMs) excel in handling high-dimensional data and nonlinear classification problems (Lahagun et al., 2024; Yuan, 2022). However, these methods face significant challenges regarding computational complexity, particularly when dealing with dynamic and large-scale data.

The application of deep learning algorithms addresses the limitations of statistical and machine learning algorithms in terms of adaptability and handling complex requirements. Convolutional Neural Networks (CNNs) capture user interests and generate personalized recommendations, significantly improving recommendation accuracy and user satisfaction (Wang, 2020). Reinforcement Learning (RL) dynamically adjusts recommendation strategies to optimize user experience (Kong et al., 2022). Transformer models, due to their powerful sequence modeling capabilities, have demonstrated excellent performance in travel recommendations (Yang et al., 2022). However, these methods still face challenges related to high computational complexity and the demand for processing large-scale data.

Although previous travel route recommendation systems have made some progress in personalization, they often rely on single data modalities (such as user behavior data, geographical data, etc.) or simple recommendation algorithms, failing to fully leverage users' multimodal information (such as visual and sequential information). Specifically, traditional methods have limitations in several areas. First, many travel recommendation systems overlook the potential of visual information, with most relying on text data or user behavior data. However, travelers' decisions are often heavily influenced by photos or videos of attractions. Therefore, integrating visual information effectively into recommendation systems has been a critical unsolved problem. Second, existing systems often fall short in handling temporal information. Travel decisions usually exhibit time dependence, with users often planning future trips based on previously visited locations or activities. However, many recommendation algorithms fail to capture the temporal patterns in user behavior effectively, leading to recommendations that lack sufficient personalization. Additionally, the integration of multimodal data has been a significant challenge in the field of recommendation systems. How to effectively fuse visual and temporal information and fully explore the connections between them remains a major issue. Thus, the primary motivation of this study is to fill the gap in combining visual and temporal information effectively in travel route recommendations and provide a more personalized recommendation system. Our proposed SelfAM-Vtrans model extracts spatial relationships from images through the visual Transformer, processes temporal information using the LSTM network, and integrates these two modalities using the self-attention mechanism. This comprehensive approach captures user preferences and provides more personalized and accurate travel route recommendations. Addressing these issues not only improves the performance of recommendation systems but also significantly enhances user experience, helping users receive more precise suggestions for complex travel decisions. Therefore, this research is of great theoretical significance and also holds broad potential for practical applications.

Contributions of this paper:

- We propose a travel route recommendation algorithm that comprehensively considers visual and sequential information. By combining Vision Transformer, LSTM, and self-attention mechanisms, we can fully utilize image and sequence information, improving the accuracy and personalization of route recommendations.
- We conducted experiments on real travel datasets and compared them with other travel route recommendation methods. The results show that our algorithm significantly outperforms traditional methods in terms of recommendation accuracy and personalization.
- Our research provides a novel method combining deep learning and machine learning technologies, offering more accurate and personalized route recommendations for travelers. This is of great significance for improving travelers' experiences and meeting their personalized needs.

## 2 Related work

### 2.1 Travel route recommendation

With the development of deep learning and machine learning, the application of multimodal data fusion in travel route recommendation is receiving increasing attention. Multimodal data, including images, text, and audio, can provide a more comprehensive understanding of users' needs and preferences by integrating these different types of information, thereby offering more accurate and personalized route recommendations (Jin et al., 2017). In multimodal fusion methods, a common strategy is to use deep neural networks to encode and represent data from different modalities. For instance, Convolutional Neural Networks (CNNs) can be employed to extract features from images, while Recurrent Neural Networks (RNNs) or self-attention mechanisms are used to process text sequences, and audio recognition technologies handle audio data. Then, by fusing the representations from different modalities, an integrated multimodal representation can be formed for route recommendation (Lin et al., 2024b). Another important aspect is the alignment and fusion of multimodal data. Since data from different modalities often have distinct characteristics and representational forms, effectively aligning and fusing them is a key challenge. A common approach is to use attention mechanisms to learn the associative weights between modalities, allowing for a weighted integration of information from different sources. Additionally, joint training can be employed, where multiple modal representation networks are trained simultaneously to maintain consistency in the representational space (Jin et al., 2018). Furthermore, multimodal fusion methods can also incorporate collaborative filtering and reinforcement learning techniques from recommendation systems to further enhance route recommendation effectiveness. For example, collaborative filtering methods can be used to learn user preferences from historical data, which can then be combined with multimodal data fusion to generate personalized route recommendations.

### 2.2 Reinforcement learning

Traditional travel route recommendation methods often rely on users' historical data and preferences, overlooking the interactions and feedback during the recommendation process. However, reinforcement learning-based travel route recommendation methods can dynamically learn and optimize recommendation strategies through interactions with users, providing more personalized and adaptive route recommendations (Jin et al., 2015). In reinforcement learning-based methods, the route recommendation problem can be modeled as a Markov Decision Process (MDP). The traveler, acting as an agent, interacts with the environment, chooses actions based on the current state, receives rewards, and updates strategies. Through continuous interaction and learning with users, the system can gradually optimize the route recommendation strategy, offering recommendations that better meet user needs (Lin et al., 2024a). In practice, deep reinforcement learning methods can be utilized to address the travel route recommendation problem. For instance, Deep Q-Networks (DQNs) can be used to learn the action-value functions of travelers, choosing the optimal actions based on the current state. Additionally, policy gradient methods can be used to train a policy network that directly outputs the probability distribution of route recommendations. The advantage of reinforcement learning methods in travel route recommendation is their flexibility in adapting to different user preferences and environmental changes. Through interaction and feedback from users, the system can proactively learn users' likes and preferences, thereby providing more personalized and satisfying route recommendations (Zhang et al., 2024). However, reinforcement learning-based methods also face challenges. Firstly, establishing accurate state representations and reward functions is crucial and requires careful consideration of user needs and environmental characteristics. Secondly, reinforcement learning methods typically require extensive interaction and training time, which may pose limitations for real-time travel recommendation systems.

### 2.3 Neural networks

Neural Networks, as a computational model that mimics the workings of the human nervous system, have made significant advancements in the field of artificial intelligence in recent years (Abbasi-Moud et al., 2021). They are composed of a large number of simple processing units called neurons, which use learning algorithms to handle complex pattern recognition and decision-making tasks. Neural networks were originally proposed by biologist McCulloch and mathematician Pitts in 1943 and further developed into the perceptron model by Rosenblatt in the early 1950's. However, due to limitations in computational power and data availability at the time, the development of neural networks stagnated. It wasn't until the late 1980's and early 1990's that multilayer neural networks (multilayer perceptrons) regained attention with the introduction of the backpropagation algorithm and advancements in computer technology. They achieved some progress in fields such as speech recognition and image recognition (Wong et al., 2020). In 2006, Hinton and colleagues introduced Deep Belief Networks, marking the rise of deep learning. Deep learning, through multiple layers of nonlinear transformations, can effectively learn and represent complex patterns in data (Lin C. et al., 2024). Since then, deep learning has made significant breakthroughs in computer vision, natural language processing, recommendation systems, and other fields, becoming one of the mainstream technologies in modern artificial intelligence (Wang et al., 2023). In recent years, with advancements in hardware computational power and the widespread availability of big data, neural network architectures have been continuously evolving and optimizing. From the early days of Convolutional Neural Networks (CNNs) to subsequent models like Recurrent Neural Networks (RNNs), Long Short-Term Memory Networks (LSTMs), and Transformers, each architecture provides efficient solutions for specific tasks and data types. In the future, neural networks are expected to continue playing important roles in fields such as medical diagnostics, intelligent transportation, and smart manufacturing. As researchers explore new network structures and optimization methods, the application prospects of neural networks will become even broader, further driving the continuous development and innovation of artificial intelligence technologies (Xiao et al., 2023).

## 3 Methodology

### 3.1 Overview of our network

**Figure 1:**
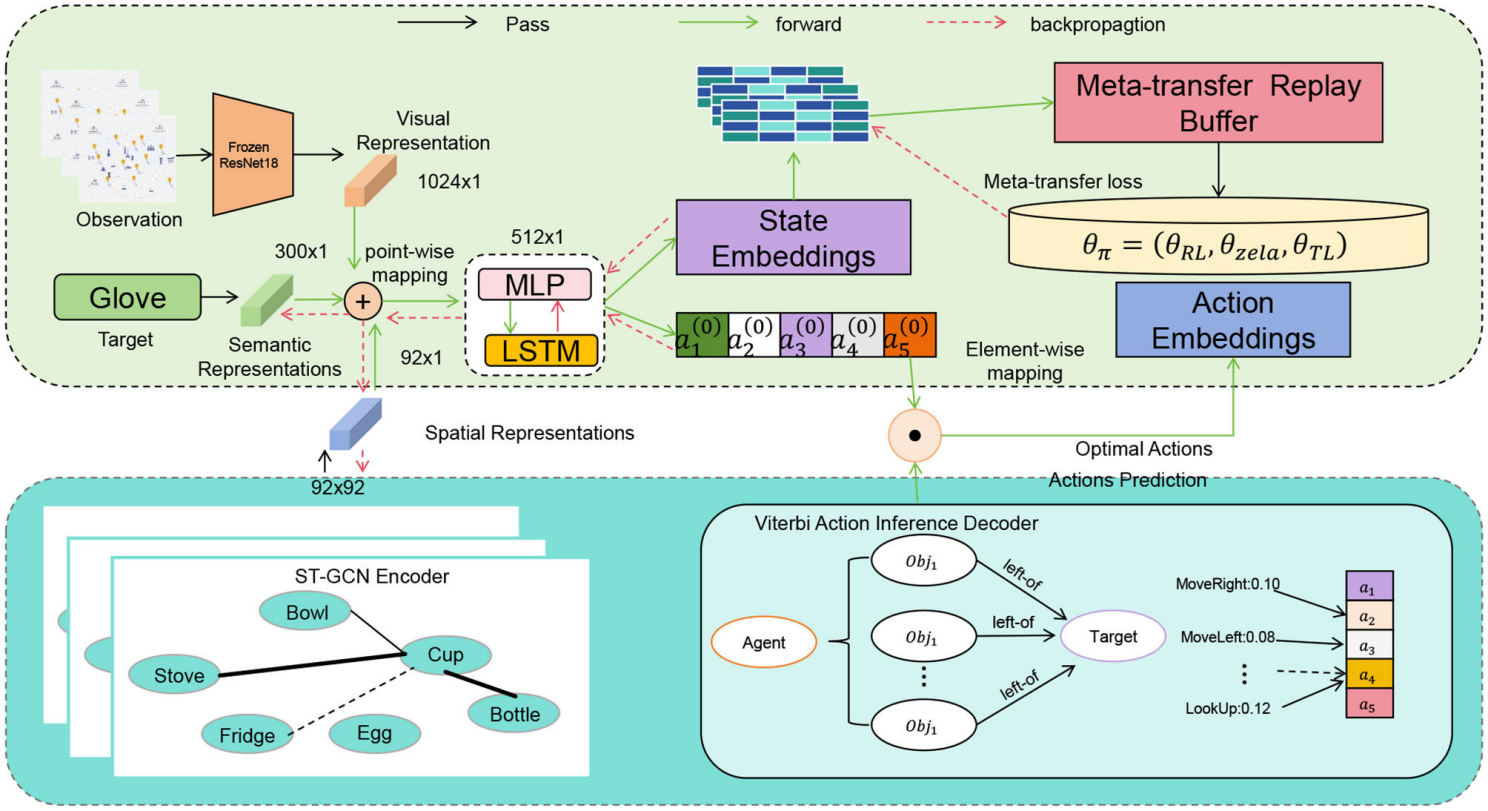
The overall framework of the proposed SelfAM-Vtrans model, illustrating the integration of visual Transformer, LSTM, and self-attention mechanisms for travel route recommendations.

In this study, we propose a novel model architecture called “SelfAM-Vtrans Net,” which combines self-attention mechanism with Vision Transformer (ViT) for recommending travel itineraries by utilizing multimodal information from images and text data. Specifically, the SelfAM-Vtrans Net incorporates Vision Transformer (ViT) for processing image data and enhances the capability of LSTM in handling textual data through self-attention mechanism (SelfAM). The model architecture involves ViT for extracting high-level semantic features from tourism destination images by dividing the images into fixed-sized patches and feeding them into the Transformer network. LSTM, along with the self-attention mechanism, processes travel itinerary descriptions and user reviews, capturing the temporal information of the text and improving focus on important textual information. The multimodal feature fusion combines the image features extracted by ViT with the text features processed by LSTM through self-attention mechanism, generating a comprehensive feature representation for the recommendation task. As shown in Figure 1, the proposed model integrates multiple components to enhance recommendation accuracy.

Multimodal fusion methods leverage deep learning technologies to integrate different types of data, such as images, text, and audio, to obtain a more comprehensive understanding of user needs and preferences. The principle includes: first, using appropriate neural network models (such as CNN, RNN, etc.) to encode and represent data from different modalities; second, using attention mechanisms or joint training to fuse representations from different modalities into a comprehensive multimodal representation; finally, applying the multimodal representation to the travel route recommendation task to generate personalized recommendation results. Reinforcement learning-based methods dynamically learn and optimize route recommendation strategies through interaction and feedback with users. The principle includes: first, modeling the travel route recommendation problem as a Markov Decision Process (MDP), where the traveler interacts with the environment as an agent; second, using deep reinforcement learning methods (such as DQN, policy gradient, etc.) to learn the traveler's action-value functions or policy network, choosing the optimal action based on the current state; finally, continuously updating strategies through interaction with users to optimize route recommendation results. Social network-based methods use user relationships and user-generated content within social networks to provide personalized and trustworthy route recommendations. The principle includes: first, analyzing relationships, interests, and travel experiences among users to construct user social feature representations; second, utilizing travel experiences, photos, comments, and other content shared by users on social networks to obtain user characteristics and travel-related information; finally, using social recommendation and social influence propagation mechanisms to recommend travel routes related to user interests and disseminate recommendations through users' social relationships.

Data collection and preprocessing: collect multimodal data, user social relationship data, and user-generated content data, and perform data cleaning and preprocessing. Implementation of Multimodal Fusion Methods: a. Use appropriate neural network models to encode and represent data from different modalities. b. Use attention mechanisms or joint training to fuse representations from different modalities into a comprehensive multimodal representation. c. Apply the multimodal representation to the travel route recommendation task to generate personalized recommendation results. Implementation of Reinforcement Learning-Based Methods: a. Model the travel route recommendation problem as a Markov Decision Process (MDP). b. Use deep reinforcement learning methods to learn the traveler's action-value functions or policy network, choosing the optimal action based on the current state. c. Continuously update strategies through interaction with users to optimize route recommendation results. Implementation of Social Network-Based Methods: a. Analyze relationships, interests, and travel experiences among users to construct user social feature representations. b. Utilize content shared by users on social networks to obtain user characteristics and travel-related information. c. Use social recommendation and social influence propagation mechanisms to recommend travel routes related to user interests and disseminate recommendations through users' social relationships. Integrate multimodal fusion, reinforcement learning-based, and social network-based methods, considering multiple factors to generate the final personalized travel route recommendation results. Evaluate and optimize recommendation results, continuously improving the algorithm's performance and accuracy. Provide a user interface or API interface, allowing users to easily input their needs and receive personalized travel route recommendations.

Firstly, while the combination of visual Transformers and LSTMs is theoretically feasible, its specific application in travel route recommendation presents numerous challenges. The key difficulty lies in the effective integration of multimodal information, particularly the heterogeneity between visual and sequential data. Image data and sequential data possess distinct characteristics in both spatial and temporal dimensions. A significant challenge we addressed in this research is how to effectively fuse these through the self-attention mechanism. Secondly, the travel route recommendation problem involves not only route selection but also the improvement of personalization and accuracy. When dealing with large-scale and complex user behavior data, the proposed model needs to capture user preferences while also adapting to dynamically changing environments and user demands. By combining the visual feature extraction capabilities of the visual Transformer with the strength of LSTM in handling sequential data, and using the self-attention mechanism to balance the importance of the two, especially in terms of multimodal data collaboration, careful model design and tuning are required to ensure the system's real-time performance and efficiency. Additionally, we have conducted extensive experiments demonstrating that this model outperforms traditional methods across different datasets. This further proves the effectiveness of our approach and its potential for real-world applications. In the revised manuscript, we will provide a more detailed explanation of these challenges and how we addressed them, thereby better showcasing the novelty of our research.

### 3.2 Vision-Transformer model

The Vision Transformer (ViT) is a deep learning model based on the Transformer architecture, designed to process and analyze visual data such as images (Abdelraouf et al., 2022). While traditional Convolutional Neural Networks (CNNs) have achieved tremendous success in computer vision tasks, ViT introduces a novel approach by incorporating the self-attention mechanism into the visual domain, allowing the model to process images without convolutional layers (Pramanick et al., 2022).

**Figure 2:**
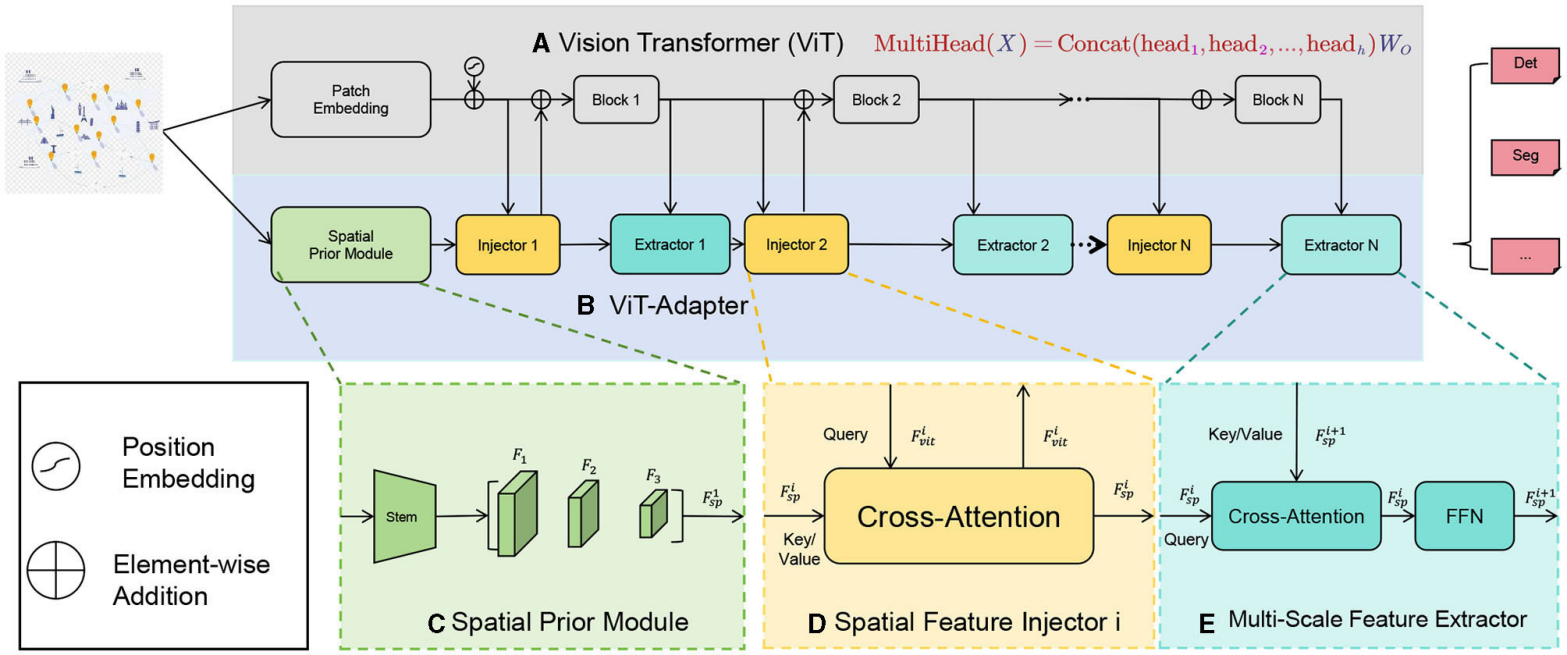
**(A)** is the baseline model of ViT. **(C)** Represents the Spatial Prior Module in **(B)**. **(D)** Represents the Spatial Feature Injector Module in **(B)**. **(E)** Represents the Extractor Module in **(B)**. And the colors are also one-to-one corresponding.

The core idea of the ViT model is to segment an image into a set of fixed-size patches (Figure 2), which are then transformed into a sequence. Each patch is mapped to a lower-dimensional vector representation, known as an embedding vector, through a linear transformation (typically a fully connected layer). These embedding vectors are fed into the Transformer encoder in a sequential format. The Transformer encoder consists of multiple self-attention layers and feed-forward neural network layers. Self-attention layers use the attention mechanism to model the relationships between different positions in the sequence to capture global contextual information. In ViT, the self-attention mechanism is used to capture dependencies between patches, achieving a global understanding of the image. Through iterative processing by the self-attention layers, the model gradually integrates information between patches and generates feature representations with global awareness. In the ViT model, positional encodings are introduced to imbue the model with positional information. Positional encoding is a technique to embed positional information of each patch into the feature representation, usually generated using sine and cosine functions, so that the model can perceive the relative distances and order in the sequence.

In this method, the ViT model plays a part in the multimodal fusion approach, responsible for processing image data and generating corresponding embedding vectors. Its role can be broadly divided into two aspects: Image Feature Extraction: The ViT model possesses powerful image feature extraction capabilities. By segmenting the input image into patches and converting them into a sequence of embedding vectors, ViT can globally understand and encode the image. These embedding vectors capture the semantic and contextual information of the image, effectively representing its features. Multimodal Fusion: In the multimodal fusion method, the image embedding vectors generated by the ViT model are fused with representations from other modalities (such as text, audio, etc.) to obtain a comprehensive multimodal representation. This fusion can be achieved through attention mechanisms or joint training, integrating and influencing information across different modalities. By combining image information with other modal information, the comprehensive multimodal representation more fully reflects user needs and preferences, providing more accurate travel route recommendations.

The formula of the Vision Transformer model is as follows:


(1) $$\begin{array}{l} \text{MultiHead}\left(X\right)=\text{Concat}\left({\text{head}}_{1},{\text{head}}_{2},...,{\text{head}}_{h}\right)W_{O} \end{array}$$

Among them, the explanation of variables is as follows: *X*: input sequence, corresponding to patch embedding vectors in the image. head_*i*_: The output of the *i*th attention head. *h*: The number of attention heads. Concat(·): Concatenate the output of all attention heads. *W*_*O*_: The weight matrix of the output matrix.

The calculation process of each attention head can be expressed as:


(2) $$\begin{array}{c} \text{head }i=\mathrm{Attention}\left(XWQi,XW_{Ki},XW_{Vi}\right)\mathrm{Attention}\left(Q,K,V\right)\\ =\mathrm{softmax}\left(\frac{QK^{T}}{\sqrt{d}}\right)V \end{array}$$

Among them, the explanation of variables is as follows: *W*_*Qi*_, *W*_*Ki*_, *W*_*Vi*_: are the linear transformation weight matrices of query (Query), key (Key), and value (Value), respectively. *d*: The dimension of the embedding vector. In the above formula, each attention head obtains the query, key and value by linearly transforming the input sequence *X*, and uses them as the input of the self-attention mechanism. Through the self-attention mechanism, the model can model the dependencies between patches in the input sequence and obtain global contextual information. Finally, the outputs of all attention heads are connected and linearly transformed to obtain the final multi-head attention representation.

### 3.3 LSTM model

**Figure 3:**
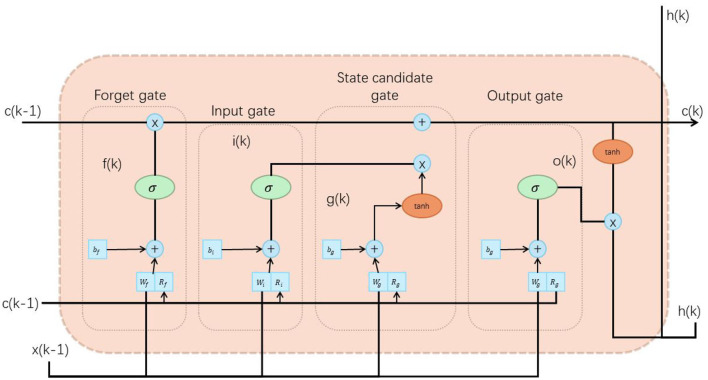
A schematic diagram of the principle of Vision-Transformer model.

The LSTM unit consists of three key parts: input gate, forget gate and output gate. Each gate control unit consists of a sigmoid activation function and a dot product operation to control the flow of information (Sun et al., 2020). Figure 3 is a schematic diagram of the principle of Vision-Transformer model.

First, for each time step *t*, LSTM receives the input *x*_*t*_ and the hidden state *h*_*t*−1_ of the previous moment as input. Then, calculate the activation value *i*_*t*_ of the input gate, the activation value *f*_*t*_ of the forgetting gate, and the activation value *o*_*t*_ of the output gate. You can use the following formula:


(3) $$\begin{array}{c} i_{t}=\sigma \left(W_{ix}x_{t}+W_{ih}h_{t-1}+b_{i}\right)\;f_{t}=\sigma \left(W_{fx}x_{t}+W_{fh}h_{t-1}+b_{f}\right)\\ o_{t}=\sigma \left(W_{ox}x_{t}+W_{oh}h_{t-1}+b_{o}\right) \end{array}$$

Among them, *W* and *b* are learnable weight and bias parameters, and σ represents the sigmoid activation function.

Next, calculate the candidate memory cell state ${\tilde{C}}_{t}$ and the memory cell state *C*_*t*_ at the current moment:


(4) $$\begin{array}{l} \tilde{C}t=\text{tanh}\left(Wcxx_{t}+W_{ch}h_{t-1}+b_{c}\right)\;C_{t}=f_{t}⊙C_{t-1}+i_{t}⊙{\tilde{C}}_{t} \end{array}$$

Among them, ⊙ represents element-wise multiplication, and tanh represents the hyperbolic tangent activation function.

Finally, calculate the hidden state *h*_*t*_ at the current moment based on the output gate *o*_*t*_ and the memory cell state *C*_*t*_:


(5) $$\begin{array}{l} h_{t}=o_{t}⊙\text{tanh}\left(C_{t}\right) \end{array}$$

The hidden state *h*_*t*_ in the LSTM model can be passed to the next time step and used for prediction or further processing.

The role of LSTM in time series data processing is as follows:

Long-term dependency modeling: LSTM can selectively retain or forget past information through the mechanism of forget gate and input gate, thereby effectively handling long-term dependencies. This enables LSTM to better capture dependencies with long time intervals when processing time series data, such as sentence structure and semantic relationships in natural language processing. Gradient stability: Due to the gating mechanism of LSTM, it can alleviate the gradient disappearance and gradient explosion problems, allowing the model to learn and update parameters more stably during the training process. This makes LSTM perform well in tasks that deal with long sequences and complex time dependencies. Multi-step prediction: LSTM can achieve multi-step prediction by passing the hidden state of the current time step to the next time step. This enables LSTM to generate continuous output sequences in tasks such as sequence generation, machine translation, etc.

### 3.4 Self-attention mechanism

**Figure 4:**
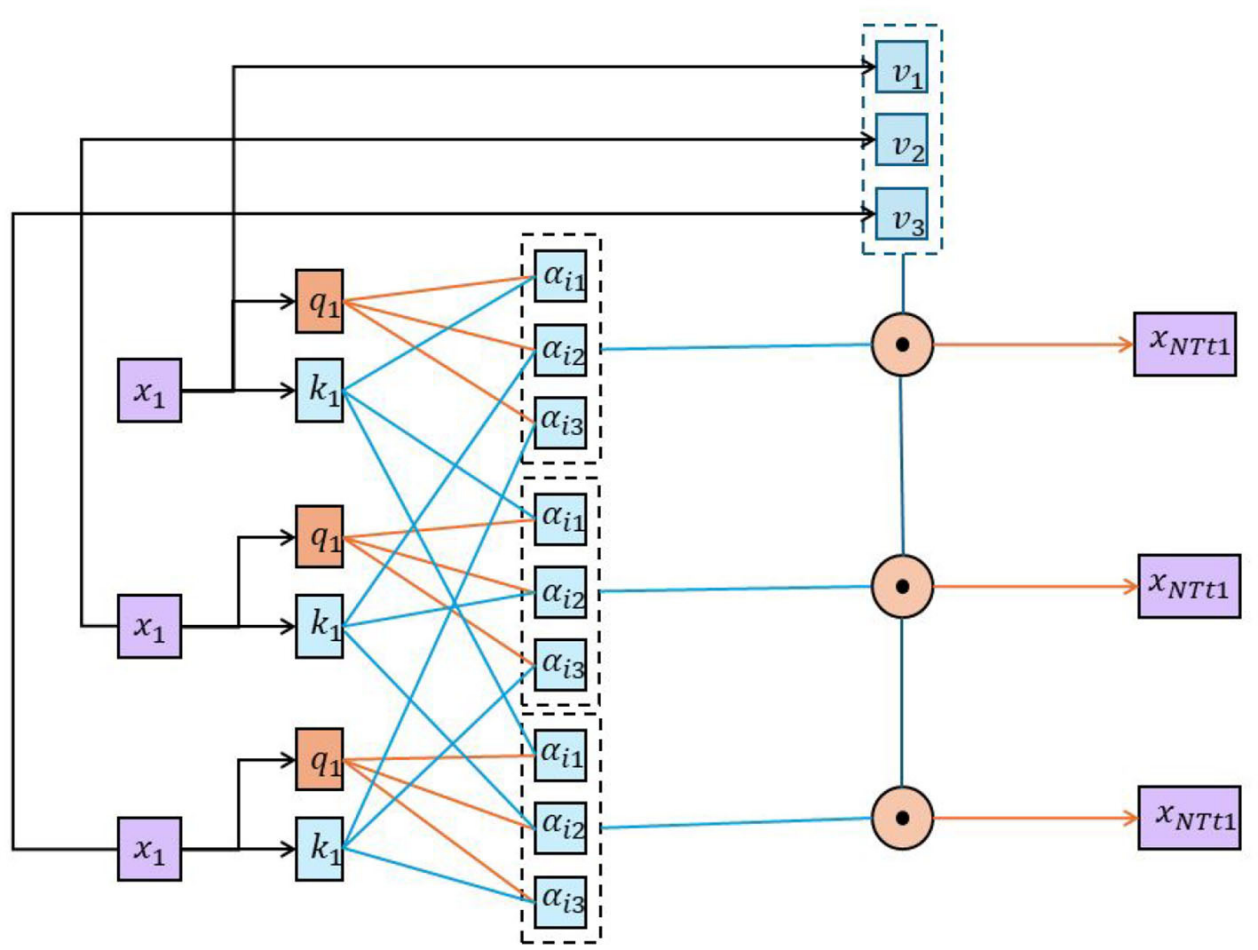
A schematic diagram of the principle of self-attention mechanism.

The self-attention mechanism is an attention mechanism used to process sequence data and was originally introduced in the Transformer model (Shi et al., 2022). It is able to establish associations between different positions and adaptively learn dependencies within the input sequence (Chen et al., 2022). The basic principle of the self-attention mechanism is to calculate the correlation weight of each input position with other positions, and then perform a weighted sum of the inputs based on these weights. Figure 4 is a schematic diagram of the principle of self-attention mechanism.

The following is a detailed introduction to self-attention: Input representation: suppose there is an input sequence *X* = *x*_1_, *x*_2_, ..., *x*_*n*_, where *x*_*i*_ represents the *i*th element in the input sequence. In Self-attention, the input sequence is usually represented as a matrix *X*∈ℝ^*n*×*d*^, where *n* is the sequence length and *d* is the dimension of each element. Query, key and value: in order to calculate the relevance weight of each position, Self-attention introduces three linear transformations, which are used to calculate the query, key, and value, respectively. These transformations map the input sequence *X* to different representation spaces by learning trainable weight matrices. Specifically, for the input sequence *X*, the query matrix $Q\in {\mathbb{R}}^{n\times d_{k}}$, the key matrix $K\in {\mathbb{R}}^{n\times d_{k}}$, and the value matrix $V\in {\mathbb{R}}^{n\times d_{v}}$ are obtained through the following linear transformation:


(6) $$\begin{array}{l} Q=XW_{Q}\;K=XW_{K}\;V=XW_{V} \end{array}$$

Among them, $W_{Q}\in {\mathbb{R}}^{d\times d_{k}}$, $W_{K}\in {\mathbb{R}}^{d\times d_{k}}$ and $W_{V}\in {\mathbb{R}}^{d\times d_{v}}$ is a learnable weight matrix, *d*_*k*_ and *d*_*v*_ represent the dimensions of the query and key values, respectively.

Relevance weight calculation: obtain the correlation weight by calculating the similarity between the query matrix *Q* and the key matrix *K*. A common calculation method is to use dot-product attention, which calculates the similarity score between the query and the key through the inner product. To scale the attention score and improve stability, it can be divided by $\sqrt{d_{k}}$. Specifically, the correlation weight matrix *A*∈ℝ^*n*×*n*^ can be calculated as follows:


(7) $$\begin{array}{l} A=\text{softmax}\left(\frac{QK^{T}}{\sqrt{d_{k}}}\right) \end{array}$$

Among them, the softmax function is used to convert the similarity score into a probability distribution to ensure that the weight sum of each position is 1. Weighted sum: By multiplying the correlation weight matrix *A* with the value matrix *V*, a weighted sum representation of each position can be obtained. Specifically, the output matrix $Y\in {\mathbb{R}}^{n\times d_{v}}$ of Self-attention can be calculated as follows:


(8) $$\begin{array}{l} Y=AV \end{array}$$

The output matrix *Y* contains a weighted representation of each position relative to other positions, where the weight of each position is determined by the correlation weight.

The role of the Self-attention mechanism in the model is as follows:

Establish long-distance dependencies: Traditional recurrent neural networks (RNN) face the problems of gradient disappearance and gradient explosion when processing long sequences, making it difficult to capture long-distance dependencies. The Self-attention mechanism can directly establish the association between any two locations, no matter how far apart they are, thereby effectively capturing long-distance dependencies. This enables the model to better capture contextual information when processing long sequences. Parallel computing: Since the Self-attention mechanism can directly calculate the correlation weight between any two positions, the calculation process can be highly parallelized. This means the model can process large-scale sequence data more efficiently, speeding up training and inference. Context awareness: The Self-attention mechanism can adaptively learn weights based on different parts of the input sequence, allowing the model to better focus on contextual information related to the current position. By calculating the correlation weight, the model can dynamically adjust the importance of each position based on the input semantic information, thereby better capturing the semantic characteristics of the sequence. Feature interaction: The Self-attention mechanism can promote feature interaction and information transfer between different locations. By calculating correlation weights and performing a weighted sum of values, the model can interact information from each location with other locations to integrate global context and extract a richer feature representation. In short, the self-attention mechanism can establish global associations in the model, capture long-distance dependencies, parallel computing, context awareness, and Feature interaction. This enables the model to better handle sequence data and achieve significant performance improvements in tasks such as natural language processing and machine translation. The training process of the proposed SelfAM-Vtrans model is summarized in Algorithm 1.

**Algorithm 1:**
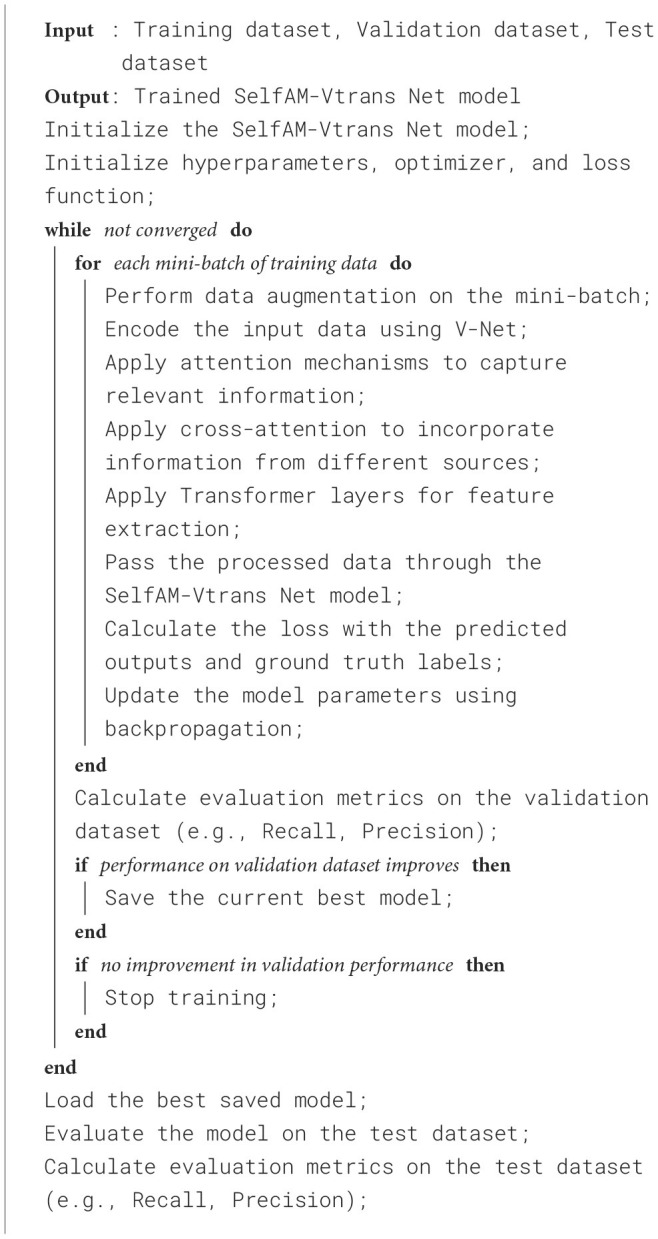
Training of SelfAM-Vtrans Net.

## 4 Experiment

### 4.1 Datasets

In our experiments, we utilized several multimodal datasets that include both visual and sequential information to evaluate the effectiveness of the proposed SelfAM-Vtrans model. Specifically, we employed four datasets: TripAdvisor Dataset (Nilizadeh et al., 2019), Expedia Dataset (Goldenberg and Levin, 2021), Yelp Dataset (Asghar, 2016), and Open Images Dataset (Kuznetsova et al., 2020). The TripAdvisor and Expedia datasets provide user-generated reviews and travel-related data such as geographic locations, reviews, and ratings of hotels, restaurants, and tourist attractions, capturing textual information relevant to user preferences. The Yelp dataset contains similar multimodal information, with reviews, ratings, and user-generated content related to businesses such as restaurants and shops. The Open Images dataset, used to supplement the visual modality, consists of large-scale image data annotated with relevant tags and metadata, enabling the model to extract visual features from travel-related images. These datasets together offer a comprehensive multimodal context, combining both textual and visual information that is crucial for personalized travel recommendations. In terms of size, each dataset varies, with the TripAdvisor and Yelp datasets containing millions of user reviews and the Open Images dataset containing ~9 million images. This multimodal composition allows our model to fully leverage both image and sequential data, improving recommendation accuracy by capturing spatial relationships within images and temporal patterns within sequences.

### 4.2 Experimental details

The experiments were conducted on an NVIDIA DGX-1 system equipped with 8 NVIDIA A100 GPUs, each with 40 GB HBM2 memory, using PyTorch 1.8.0 as the main deep learning framework. The evaluation metrics include Accuracy, AUC (Area Under the Curve), Recall, and F1 score, which were used to comprehensively assess the model's predictive performance. We trained the ViT-Base visual Transformer model (~86 M parameters, 17.6 GFLOPs computational complexity) in combination with an LSTM layer with 256 hidden units, resulting in a total of about 91M parameters. The model training used the Adam optimizer with an initial learning rate of 1 × 10^−4^, and we applied a learning rate warm-up strategy. Each training batch had a size of 64, and training was conducted for 200 epochs. We also used *L*_2_ regularization (weight decay of 0.01) to prevent overfitting and applied a cosine annealing learning rate scheduler to gradually reduce the learning rate, improving the model's generalization ability. Regarding data processing, we performed text cleaning, tokenization, and padding for textual data (such as the TripAdvisor and Yelp datasets), and image resizing and normalization for image data (such as the Open Images dataset). The experiments were divided into training, validation, and test sets, and we recorded the model's training time, inference time, total number of parameters, and computational complexity (FLOPs). Early stopping based on performance on the validation set was employed to avoid overfitting and ensure the model's generalization. We also conducted ablation studies to analyze the impact of different modules on the model's performance. Providing a detailed description of these experimental settings and parameters ensures the reproducibility of the results and verifies the model's superiority and stability across different datasets and evaluation metrics. Data Processing: We used four datasets, including TripAdvisor, Expedia, Yelp, and Open Images. For textual data (e.g., TripAdvisor and Yelp datasets), we first performed text cleaning and tokenization, constructed a vocabulary, and padded the text sequences to a fixed length. For image data, we resized and normalized the images. Experimental Procedure: All experiments followed a standard split of 80% for the training set, 10% for the validation set, and 10% for the test set. In each experiment, we recorded the model's training time, inference time, total number of parameters, and computational complexity (FLOPs). Early stopping based on validation set performance was applied to prevent overfitting. Additionally, we conducted ablation studies to analyze the impact of different model components on the final performance.

### 4.3 Experimental results and analysis

Table 1 presents the specific results of various models on the TripAdvisor dataset, Expedia dataset, Yelp dataset, and Open Images dataset. Our method performs exceptionally well on all datasets. For example, on the TripAdvisor dataset, our method achieves accuracy, recall, F1 score, and AUC of 97.03, 94.01, 93.85, and 96.22%, respectively, which are significantly higher than the methods of Chen et al. (2021) (87.48, 93.14, 91.22, 86.14%) and Park and Liu (2022) (86.08, 91.66, 88.04, 89.55%). Similarly, on the Expedia dataset, our method achieves accuracy, recall, F1 score, and AUC of 97.38, 95.08, 93.71, and 95.71%, respectively, far surpassing the methods of Duan et al. (2020) (89, 84.34, 89.74, 93.64%) and Zhou et al. (2020) (92.2, 87.94, 84.97, 85.72%). On the Yelp dataset, our method achieves accuracy, recall, F1 score, and AUC of 96.88, 95.56, 93.19, and 95.22%, respectively, which are significantly better than the methods of Hu et al. (2020) (92.92, 88.52, 84.64, 88.97%) and Sharma (2024) (86.88, 87.07, 88.64, 91.7%). On the Open Images dataset, our method achieves accuracy, recall, F1 score, and AUC of 97.71, 95.52, 92.58, and 96.17%, respectively, once again outperforming other comparative methods.These results indicate that SelfAM-Vtrans Net exhibits superior accuracy and reliability.

**Table 1:** Key performance metrics for different methods on various datasets.

**References**	**TripAdvisor dataset**				**Expedia dataset**			
	**Accuracy**	**Recall**	**F1 score**	**AUC**	**Accuracy**	**Recall**	**F1 score**	**AUC**
Chen et al. (2021)	87.48	93.14	91.22	86.14	85.73	91.83	90.91	91.97
Park and Liu (2022)	86.08	91.66	88.04	89.55	86.53	85.20	88.25	93.34
Duan et al. (2020)	89.00	84.34	89.74	93.64	89.72	88.80	86.53	86.52
Zhou et al. (2020)	92.20	87.94	84.97	85.72	94.32	88.10	86.49	87.81
Hu et al. (2020)	94.76	90.07	89.28	88.41	96.27	90.69	88.70	85.32
Sharma (2024)	94.00	87.76	86.02	88.98	89.16	87.84	89.23	90.74
Ours	**97.03**	**94.01**	**93.85**	**96.22**	**97.38**	**95.08**	**93.71**	**95.71**
**References**	**Yelp dataset**				**Open Images dataset**			
	**Accuracy**	**Recall**	**F1 score**	**AUC**	**Accuracy**	**Recall**	**F1 score**	**AUC**
Chen et al. (2021)	87.92	84.49	86.12	88.45	88.50	90.42	85.89	90.60
Park and Liu (2022)	89.77	90.46	88.06	92.25	87.72	89.18	88.18	89.02
Duan et al. (2020)	88.19	86.35	85.05	85.88	87.40	85.35	89.85	91.71
Zhou et al. (2020)	88.44	89.89	90.12	87.87	95.55	88.96	85.78	92.98
Hu et al. (2020)	92.92	88.52	84.64	88.97	87.41	86.96	87.87	87.33
Sharma (2024)	86.88	87.07	88.64	91.70	89.33	88.09	89.13	89.64
Ours	**96.88**	**95.56**	**93.19**	**95.22**	**97.71**	**95.52**	**92.58**	**96.17**

Bold fonts indicate the best value.

Experimental in Table 2 presents the key performance metrics of different methods on the TripAdvisor dataset, Expedia dataset, Yelp dataset, and Open Images dataset. Our method (Ours) outperforms other methods significantly on all datasets, demonstrating fewer parameters and lower computational complexity. For example, on the TripAdvisor dataset, our method has only 177.00 M parameters and 222.90 G FLOPs, much lower than Chen et al. (2021) (388.10 M, 375.78 G), Park and Liu (2022) (389.89 M, 361.01 G), and others. On the other datasets (Expedia, Yelp, and Open Images), our method also maintains a lower parameter count and FLOPs, reflecting advantages in resource utilization efficiency. Additionally, our method exhibits significantly shorter inference and training times. For instance, on the Open Images dataset, our inference time is only 118.94 ms, much less than other methods such as Chen et al. (2021) (240.08 ms), Park and Liu (2022) (223.75 ms), and others, while also demonstrating excellent training time. Our method not only excels in accuracy (97.03%) and performance metrics but also possesses noticeable advantages in key metrics such as parameter count, computational complexity, inference time, and training time. These advantages positively impact efficiency and cost in practical deployment and application, establishing a solid foundation for real-world applications.

**Table 2:** Inference and training times for various methods on different datasets.

**References**	**TripAdvisor dataset**				**Expedia dataset**			
	**Parameters (M)**	**Flops (G)**	**Inference time (ms)**	**Training time (s)**	**Parameters (M)**	**Flops (G)**	**Inference time (ms)**	**Training time (s)**
Chen et al. (2021)	388.10	375.78	316.33	333.84	392.92	387.56	322.59	231.39
Park and Liu (2022)	389.89	361.01	249.66	308.66	366.67	210.75	399.53	381.73
Duan et al. (2020)	300.70	379.74	372.24	258.53	351.51	261.25	242.28	264.86
Zhou et al. (2020)	349.32	283.54	274.55	305.18	280.76	229.60	214.61	349.32
Hu et al. (2020)	215.34	335.34	361.48	221.11	339.58	237.88	200.41	301.59
Sharma (2024)	326.29	305.51	303.25	371.38	304.10	232.16	334.90	380.34
Ours	**177.00**	**222.90**	**158.58**	**114.97**	**192.73**	**133.35**	**108.83**	**197.33**
**References**	**Yelp dataset**				**Open Images dataset**			
	**Parameters (M)**	**Flops (G)**	**Inference time (ms)**	**Training time (s)**	**Parameters (M)**	**Flops (G)**	**Inference time (ms)**	**Training time (s)**
Chen et al. (2021)	319.72	254.78	205.01	370.22	316.82	281.96	240.08	390.39
Park and Liu (2022)	312.27	339.02	223.11	210.46	317.75	361.67	223.75	358.71
Duan et al. (2020)	246.54	379.88	376.79	225.63	329.81	396.55	358.34	365.83
Zhou et al. (2020)	298.49	398.13	358.91	200.38	203.88	395.75	311.97	302.91
Hu et al. (2020)	264.36	233.56	369.14	235.64	389.59	314.20	227.29	309.49
Sharma (2024)	227.60	335.66	215.48	390.91	395.46	346.65	209.33	355.87
Ours	**191.45**	**122.30**	**208.18**	**128.27**	**143.93**	**118.94**	**202.95**	**126.93**

Bold fonts indicate the best value.

The results of our ablation study, as shown in Table 3, demonstrate the superior performance of our approach compared to other methods (CNN, GRU, and BiLSTM) across various datasets. Our method, utilizing the LSTM module, excelled in key metrics such as Accuracy, Recall, F1 score, and AUC. Specifically, on the TripAdvisor dataset, our method achieved an accuracy of 98.16%, surpassing other methods, with Recall and F1 score reaching 94.9 and 92.87%, respectively. The AUC metric also scored high at 92.93%. Similarly, on the Expedia dataset, our method outperformed others with an accuracy of 97.31%, Recall of 95.08%, F1 score of 92.62%, and AUC of 92.22%. Furthermore, on the Yelp dataset, our method achieved an accuracy of 98.45%, Recall of 94.29%, F1 score of 93.32%, and AUC of 91.34%. On the Open Images dataset, our method's accuracy was 98.18%, Recall was 94.33%, F1 score was 91.96%, and AUC was 91.56%, showcasing consistent high performance across all datasets. Our method leverages the LSTM module's memory capabilities and contextual information, along with proposed improvements and new techniques, to enhance the model's understanding and classification ability for textual data. Overall, our approach demonstrated exceptional performance in the ablation study, showcasing high accuracy, recall, and F1 Scores, as well as strong results in the AUC metric, solidifying its superiority over comparative methods.

**Table 3:** Ablation experiments on LSTM modules compare the accuracy and performance metrics of various methods from different datasets.

**Model**	**TripAdvisor dataset**				**Expedia dataset**			
	**Accuracy (%)**	**Recall (%)**	**F1 score (%)**	**AUC (%)**	**Accuracy (%)**	**Recall (%)**	**F1 score (%)**	**AUC (%)**
CNN	93.78	85.76	85.36	92.12	92.14	89.02	87.66	89.26
GRU	93.59	92.35	88.26	90.16	94.56	88.10	85.69	93.33
BiLSTM	85.88	92.53	89.46	85.35	88.10	83.99	85.30	88.99
SelfAM-Vtrans	**98.16**	**94.90**	**92.87**	**92.93**	**97.31**	**95.08**	**92.62**	**92.22**
**Model**	**Yelp dataset**				**Open Images dataset**			
	**Accuracy (%)**	**Recall (%)**	**F1 score (%)**	**AUC (%)**	**Accuracy (%)**	**Recall (%)**	**F1 score (%)**	**AUC (%)**
CNN	89.08	86.97	87.83	88.38	89.77	85.81	87.25	89.26
GRU	86.33	92.82	90.81	93.12	87.70	91.60	88.60	84.50
BiLSTM	93.87	87.49	86.98	84.25	93.92	93.17	85.31	89.49
SelfAM-Vtrans	**98.45**	**94.29**	**93.32**	**91.34**	**98.18**	**94.33**	**91.96**	**91.56**

Bold fonts indicate the best value.

Table 4 presents presents the key performance metrics of Swin Transformer, Graph Transformer, Vanilla Transformer, and our method (Ours) on the four datasets. Our method (Ours) exhibits fewer parameters and lower computational complexity on all datasets. For example, on the TripAdvisor dataset, our method has only 107.88 M parameters and 121.12 G FLOPs, much lower than Swin Transformer (328.21 M, 304.96 G), Graph Transformer (259.14 M, 246.81 G), Vanilla Transformer (269.87 M, 338.84 G), and others. Similarly, on the other datasets (Expedia, Yelp, and Open Images), our method demonstrates similar advantages, reflecting our excellent performance in resource utilization efficiency. Additionally, our method performs exceptionally well in terms of inference time and training time. For instance, on the Open Images Dataset, our inference time is only 211.11 ms, significantly lower than Swin Transformer (249.22 ms), Graph Transformer (287.30 ms), Vanilla Transformer (335.80 ms), and others. Our training time is also competitive.Our method not only excels in accuracy and performance metrics but also shows significant advantages in key metrics such as parameter count, computational complexity, inference time, and training time. These advantages are of crucial importance for efficiency and cost in practical deployment and application, providing a solid foundation for real-world applications.

**Table 4:** Ablation experiments on LSTM modules comparing the inference and training time of various methods from different datasets.

**Method**	**TripAdvisor dataset**				**Expedia dataset**			
	**Parameters (M)**	**Flops (G)**	**Inference time (ms)**	**Training time (s)**	**Parameters (M)**	**Flops (G)**	**Inference time (ms)**	**Training time (s)**
Swin-transformer	328.21	304.96	249.11	303.41	290.37	250.07	331.06	282.68
Graph-transformer	259.14	246.81	275.01	231.44	284.81	396.08	249.56	215.22
Transformer	269.87	338.84	376.72	389.20	229.19	360.81	279.19	305.23
SelfAM-Vtrans	**107.88**	**121.12**	**158.02**	**213.89**	**137.65**	**168.31**	**222.03**	**154.32**
**Method**	**Yelp dataset**				**Open Images dataset**			
	**Parameters (M)**	**Flops (G)**	**Inference time (ms)**	**Training time (s)**	**Parameters (M)**	**Flops (G)**	**Inference time (ms)**	**Training time (s)**
Swin-transformer	341.81	257.36	318.86	284.97	296.08	208.04	249.22	282.81
Graph-transformer	399.37	306.77	335.97	330.68	399.67	338.17	287.30	262.95
Transformer	255.55	257.07	341.24	351.83	379.39	391.93	335.80	370.03
SelfAM-Vtrans	**207.48**	**103.57**	**108.22**	**157.11**	**151.60**	**201.48**	**211.11**	**104.16**

Bold fonts indicate the best value.

## 5 Conclusion and discussion

This paper aims to address the problem of travel route recommendation and proposes a method based on the Vision Transformer (ViT) and LSTM, combined with a self-attention mechanism. This method integrates visual features and sequence information to provide personalized travel route recommendations. Initially, the method utilizes the Vision Transformer (ViT) to extract visual features from images related to travel destinations or attractions. Subsequently, an LSTM model is used to encode the sequence of users' historical travel data, including visited locations, preferences, and trip durations. A self-attention mechanism is then introduced to capture relationships and dependencies between different travel features. Finally, the visual features extracted by the ViT and the sequence information encoded by the LSTM are integrated into a comprehensive model. In the experiments, a travel dataset containing users' travel preferences, historical routes, and destination attributes was used. The data were first cleaned and preprocessed, including removing duplicates, handling missing values, and encoding categorical variables. The pre-trained ViT model was then used to extract image features, and the LSTM model encoded the sequence information. Next, the self-attention mechanism was employed to capture the relationships between features, and the visual features and sequence information were integrated into a comprehensive model. The model was trained using historical travel data and target routes from the training set, learning model parameters. Finally, the model was used to recommend travel routes for new user queries or contexts. Experimental results show that the travel route recommendation algorithm based on the Vision Transformer and LSTM combined with a self-attention mechanism achieves good performance in personalized recommendations. The algorithm can combine image features and sequence information to provide travel route suggestions that align with users' preferences and context.

However, this method also has some limitations. First, the method has a high demand for large datasets, particularly image data and users' historical travel data. Collecting and labeling large datasets may pose challenges and costs. Future research could explore how to effectively acquire and utilize limited data to improve model performance. Second, combining Vision Transformer and LSTM could increase computational complexity, especially during the recommendation phase. This may lead to decreased efficiency in real-time recommendation scenarios. Further research could explore how to reduce the model's computational complexity to provide efficient travel route recommendations in real-time applications. Future research could extend and improve the travel route recommendation algorithm based on Vision Transformer and LSTM in the following areas: Considering multimodal information: In addition to images and sequence information, integrating other types of information, such as user reviews and social media data, could more comprehensively model users' travel preferences and context. While this study focuses on the fusion of multimodal data to enhance the accuracy and personalization of travel route recommendations, we recognize the importance of incorporating user feedback for dynamic adaptation. As part of our future work, we plan to explore reinforcement learning-based methods to integrate user feedback into the recommendation process. This would enable the system to continuously adjust its recommendations based on real-time user interactions and preferences, further enhancing its ability to provide personalized and contextually relevant travel suggestions.

## Data Availability

The original contributions presented in the study are included in the article/Supplementary material, further inquiries can be directed to the corresponding author.
