# Supplementary material for: A multimodal travel route recommendation system leveraging visual Transformers and self-attention mechanisms
Source: Front Neurorobot. 2024 Nov 26;18:1439195. doi: 10.3389/fnbot.2024.1439195 (PMC11628496; doi:10.3389/fnbot.2024.1439195)
Supplement: Supplementary file 1 [file Data_Sheet_1.pdf]

Supplementary Material

1 SUPPLEMENTARY TABLES AND FIGURES

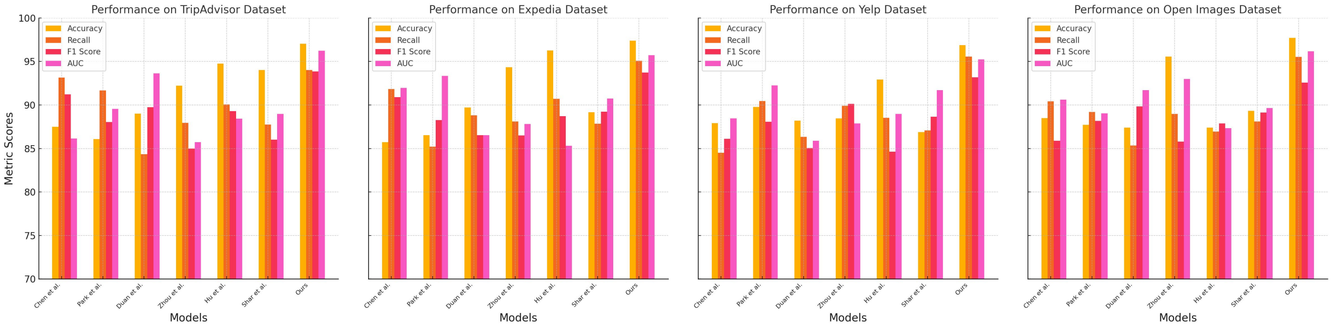

Figure S1. Key Performance Metrics for Different Methods on Various Datasets

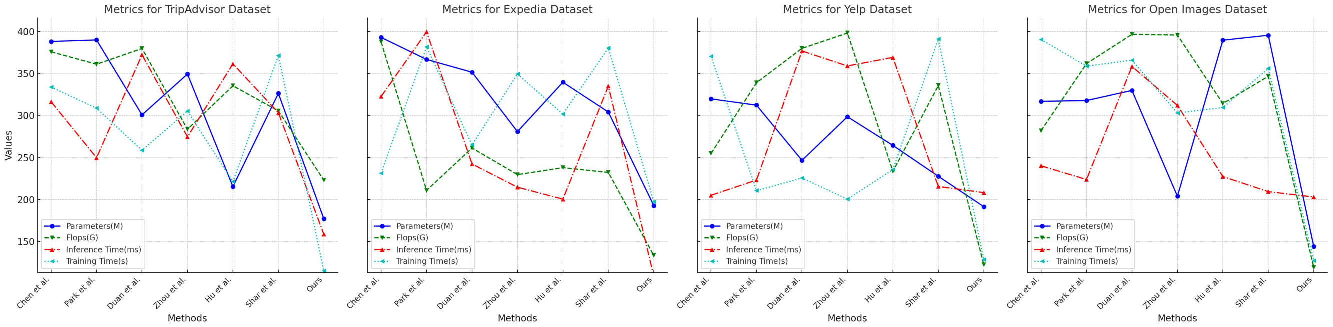

Figure S2. Inference and Training Times for Various Methods on Different Datasets

These four pictures are the visualization pictures corresponding to Tables 1, 2, 3, and 4 respectively.

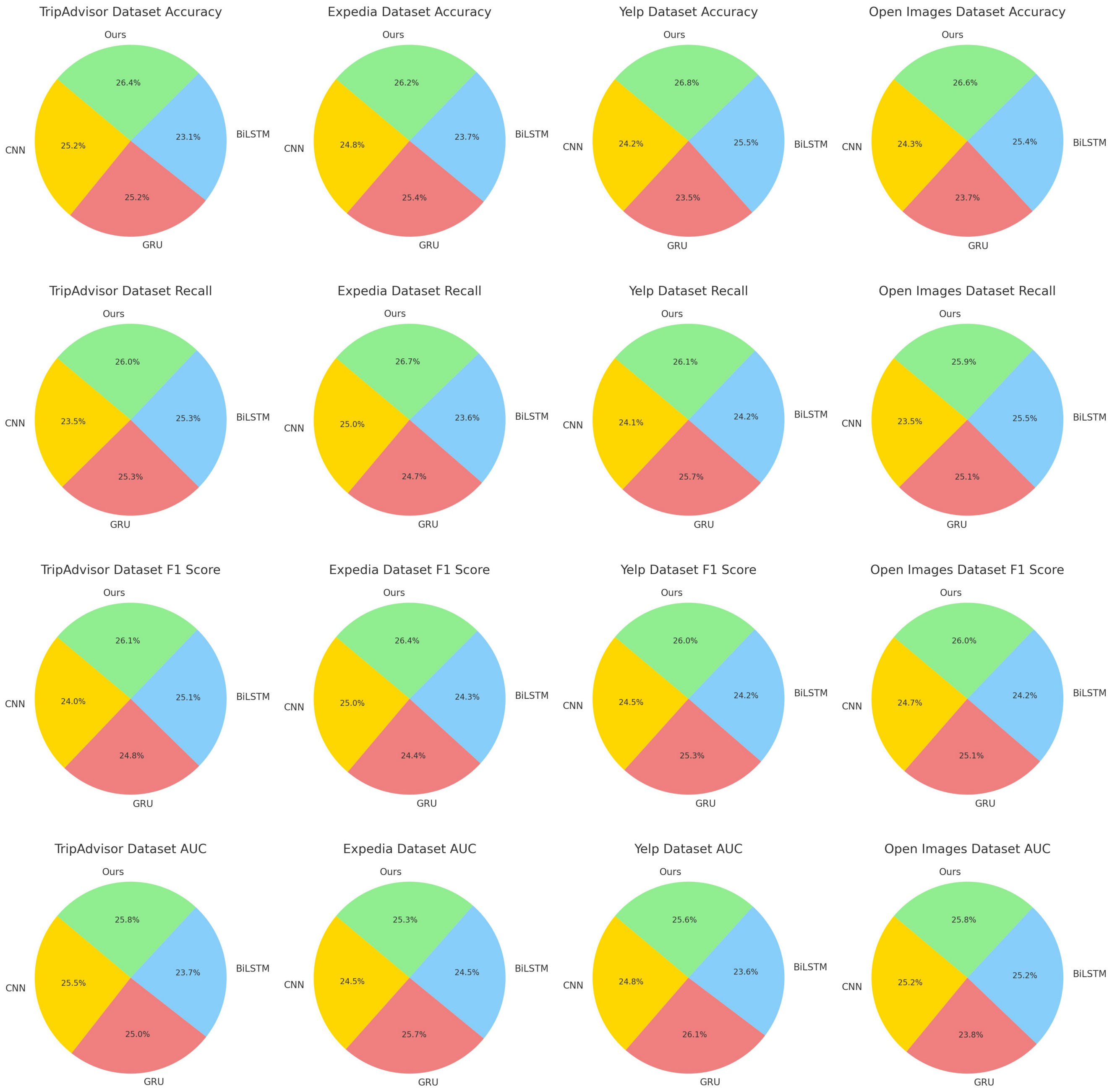

Figure S3. Ablation experiments on LSTM modules compare the accuracy and performance metrics of various methods from different datasets.

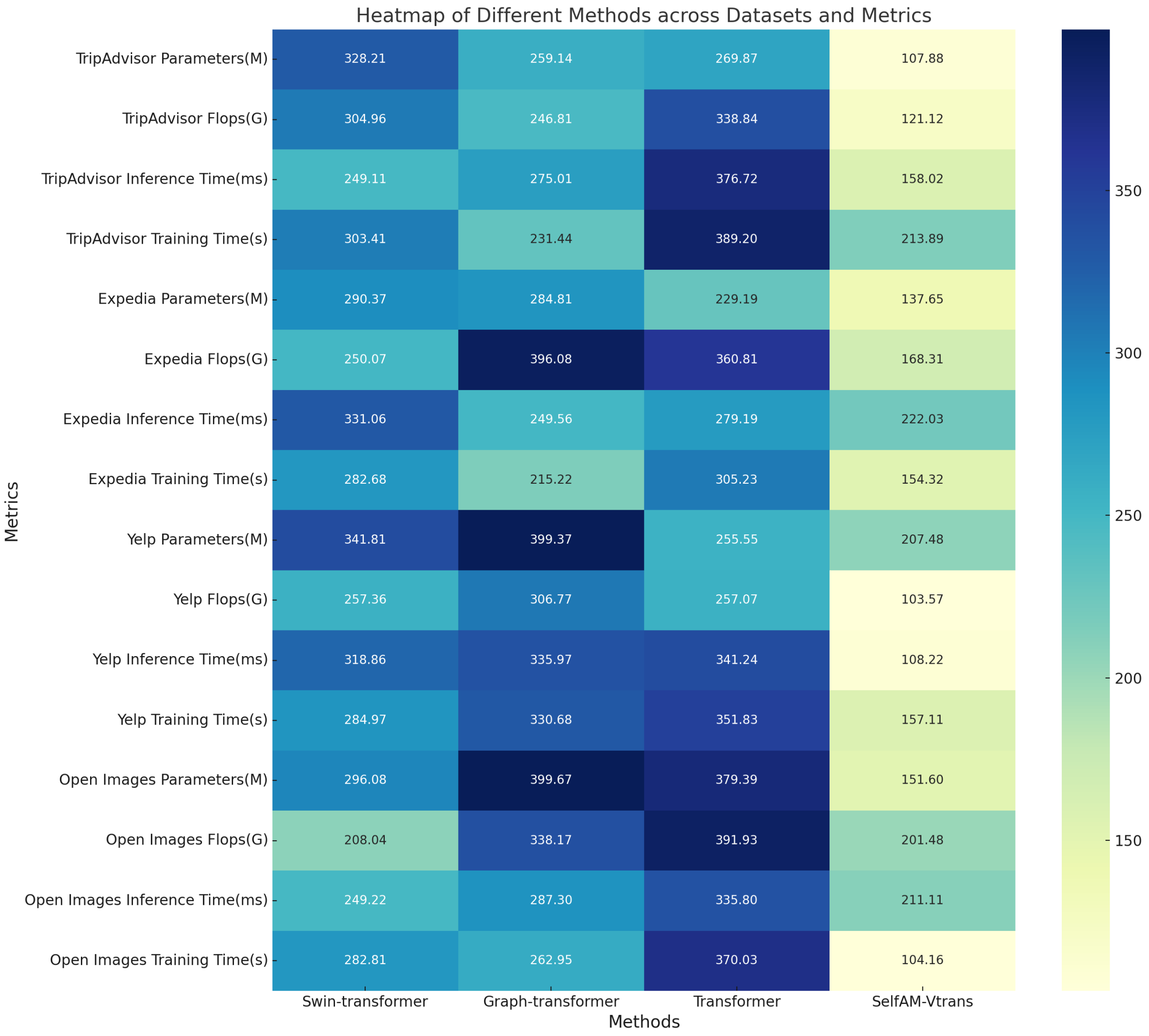

Figure S4. Ablation experiments on LSTM modules compare the inference and training time of various methods from different datasets.
